# Unactivated leukocyte expression of C-reactive protein is minimal and not dependent on rs1205 genotype

**DOI:** 10.1038/s41598-021-85272-9

**Published:** 2021-03-11

**Authors:** L. G. Best, C. Azure, K. Martell, K. S. Tsosie, B. Voels

**Affiliations:** 1 University of North Dakota, Grand Forks, ND USA; 2 Natural Sciences, Turtle Mountain Community College, Belcourt, ND USA; 3 Science, Cankdeska Cikana Community College, Fort Totten, ND USA; 4 1935 118th Ave NW, Watford City, ND 58854 USA

**Keywords:** Genetics, Immunology

## Abstract

C-reactive protein (CRP), a prominent component of the innate immune system, is implicated in the pathophysiology of many conditions. CRP production primarily occurs in the liver; but contributions from other tissues is unclear. The Genotype-Tissue Expression Portal shows essentially no expression in whole blood and reports in the literature are conflicting. Multiple genomic variants influence serum levels of CRP. We measured *CRP* mRNA expression in leukocytes and sought to determine if rs1205 genotype influences leukocyte expression. Leukocytes were obtained from 20 women differing by genotype. Quantitative, real-time PCR (RT-qPCR) detected *CRP* and reference gene (*GAPDH*) mRNA. Leukocyte expression was calculated by the 2^ΔCT^ method, and against a standard curve. Digital drop PCR was also used to calculate expression ratios. Student's t test and linear regression methods examined possible differences between genotypes. During 32 runs (10 replicates each), the RT-qPCR mean (SD) *CRP*/*GAPDH* ratio was 3.39 × 10^–4^ (SD 1.73 × 10^–4^) and 3.15 × 10^–4^ (SD 1.64 × 10^–4^) for TT and CC genotypes respectively, p = 0.76; and digital drop PCR results were similar. Serum CRP was not significantly different between genotypes, nor correlated with leukocyte expression. *CRP* is minimally expressed in unactivated leukocytes and this expression is not likely influenced by rs1205 genotype.

## Introduction

C-reactive protein (CRP) is an important component of the innate immune system^1^; and has been promoted as a non-specific measure of inflammatory status in epidemiologic studies of cardiovascular disease and other conditions^2^.

Previously we demonstrated an association between rs1205 in the 3′ untranslated region of *CRP* and severe pre-eclampsia in an American Indian cohort^3^; and subsequently provided additional evidence for this association and 2 other single nucleotide polymorphisms (SNPs) related to the *CRP* gene^4^, as have others^5^. While the primary source of circulating CRP appears to be the liver^6^, other tissues, including endothelium^7^, macrophages^8^, kidney^9^, brain^10^, and placenta^11^ are also thought to secrete CRP, although often under various stimulating conditions. Evidence for leukocyte expression of *CRP* has been contentious due to difficulties with non-specific antibody detection^12,13^, differing real-time quantitative polymerase chain reaction (RT-qPCR) protocols, as well as characteristics of the *CRP* gene that complicate primer design for RT-qPCR. The Genotype-Tissue Expression Portal (GTEx) shows essentially no expression in whole blood^14^.

The present study was undertaken to determine if *CRP* expression could be detected in unactivated leukocytes from an American Indian population; and if it is influenced by rs1205 genotype. This variant is common, with a minor allele frequency of 46% in this population^3^.

## Results

### Expression of *CRP* by unactivated leukocytes

Although there is evidence of *CRP* mRNA expression by leukocytes^12^, this is not uniformly accepted^9^ and typically not examined in the unstimulated state^15^.The ratio of *CRP* to reference gene expression in the present study was on the order of 10^–4^ by either of the four methods of calculation. The maximum value was 1.03 × 10^–3^. The distribution of both the 2^ΔCT^ and the standard curve expression ratios were Normal after excluding outlier samples as noted in Methods (1.5 X above the 75%ile). The distributions of the digital drop expression ratios were non-Normal for both the *GAPDH* and *ACTB* reference genes and were natural log transformed prior to the Student's t-test. See Table 1 for results.

**Table 1:** Comparison of means of rs1205 TT and CC genotypes.

	Rs1205 TT genotype, N = 10	Rs1205 CC genotype, N = 10	P value
Age	45.7	41.0	0.128
BMI	31.56	33.36	0.539
Tobacco use*	7/10 = 70%	4/10 = 40%	0.369
hsCRP (mg/L)**, N = 9,10	4.43	4.47	0.979
ln hsCRP, N = 9,10	1.226	1.294	0.840
**Outlier samples excluded**			
*CRP/GAPDH* expression ratio qPCR, 2^ΔCT^ Method, N = 9, 10***	3.395 × 10^–4^	3.153 × 10^–4^	0.759
*CRP/GAPDH* expression ratio qPCR, Std. curve method, N = 8, 9	3.653 × 10^–4^	3.550 × 10^–4^	0.909
*CRP/GAPDH* expression ratio Digital drop PCR, N = 10, 9	0.827 × 10^–4^	0.605 × 10^–4^	0.961****
*CRP/ACTB* expression ratio Digital drop PCR, N = 8, 10	0.136 × 10^–4^	0.097 × 10^–4^	0.599

*Smoking defined as any reported use of tobacco.

**One value excluded as > 15 SD above the mean.

***N = number of samples from TT and CC individuals respectively.

****p value for comparison of ln transformed CRP/GAPDH ratios. The means are non-transformed for ease of comparison.

No template and no reverse transcription controls were run for all samples, in both RT-PCR and digital drop methods. These showed no evidence of environmental contamination in the NTC wells and a minimum of 2^10^ (1024X) lower expression for the NRT compared to the *GAPDH* reference gene. The *CRP* NRT however, showed CT values generally indistinguishable from the samples undergoing reverse transcription. Similarly, the digital drop runs showed a minimum of 400X lower expression in NRT samples for the reference gene, as opposed to essentially equivalent expression for *CRP*.

Within the 10 wells of the RT-qPCR runs, the 2^ΔCT^ mean coefficient of variation was 8.6% (range 3.4–29.3%) among 32 independent runs, subject to the exclusion of outlier wells as detailed in methods. Similarly, within standard curve runs, the mean coefficient of variation (CV) was also 8.6% among 23 independent runs. In the digital drop runs there were no samples replicated within a run.

For three samples the 10 replicate run of the RT-qPCR assay was repeated on 7, 2 and 2 occasions, with a mean CV of 6.6% and the inter-run mean expression ratios were used in the comparisons between genotype groups. Samples were replicated an average of 2.5 and 1.5 times for digital drop GAPDH and ACTB runs respectively. The mean, inter-run CV was 32.7% for GAPDH and 16.2% for ACTB runs. In each digital drop run, a reference sample of standard BIO-RAD template concentration was analyzed for both *CRP* and the reference gene in each run. The results of these assays were as follows: *CRP* CV = 12.4% (17 runs), *GAPDH* CV = 12.1% (13 runs), *ACTB* CV = 10.7% (5 runs).

After excluding outlier samples, the correlations between the four different measures of expression were not significant, with the exception of the 2^ΔCT^ expression and the standard curve method, with a Pearson correlation of 0.572, *p* = 0.016.

### Hypothesized correlation between serum CRP and leukocyte expression

Examining the potential correlations between serum hsCRP and these measures of leukocyte expression, only the digital drop results using *GAPDH* as reference showed a significant Pearson correlation of negative 0.520, *p* = 0.027.

### Hypothesized association between genotype and leukocyte expression

Increased serum hsCRP is clearly associated with the rs1205, CC genotype in the literature ^16–18^, however there is no reported rs1205 influence on mRNA expression in any tissue^19^. Our results show essentially equivalent mean hsCRP between the two genotypes and no significant genotypic differences in mRNA expression as seen in the results of univariate and multivariate models of Table 2. The four measures of expression and serum hsCRP are given as dependent variables and the primary independent predictor is rs1205 genotype (TT = 0 vs CC = 1). Multivariate analysis was done by including age, BMI and tobacco use as covariates. These covariates were selected on the basis of demonstrated influence on hsCRP in the literature^20,21^; but BMI (standardized beta = 0.885, *p* = 0.001) and tobacco use (standardized beta = 0.417, *p* = 0.076) were shown to be predictive, even in the present, modestly-powered study.

**Table 2:** Univariate and multivariate linear regression with *CRP* expression and hsCRP as dependent variable.

	β coefficient	Standard error	Std β coefficient	P value
**Univariate models, TT = 0 vs CC = 1 genotype (outlier samples excluded)**				
*CRP/GAPDH* expression ratio qPCR, 2^ΔCT^ Method, N = 9, 10*	− 0.242	0.775	− 0.075	0.759
*CRP/GAPDH* expression ratio qPCR, Std. curve method, N = 8, 9	− 0.103	0.894	− 0.030	0.909
*CRP/GAPDH* expression ratio Digital drop PCR, N = 10, 9	− 0.222	0.234	− 0.224	0.356
*CRP/ACTB* expression ratio Digital drop PCR, N = 8, 10	− 0.040	0.042	− 0.232	0.355
hsCRP, N = 9, 10	0.040	1.488	0.007	0.979
Linear Regression model adding age, BMI, smoking**				
**Multivariate models (outlier samples excluded)**				
*CRP/GAPDH* expression ratio qPCR, 2^ΔCT^ Method, N = 9, 10	0.037	0.894	0.012	0.967
*CRP/GAPDH* expression ratio qPCR, Std. curve method, N = 8, 9	− 0.090	1.114	− 0.026	0.937
*CRP/GAPDH* expression ratio Digital drop PCR, N = 10, 9	− 0.037	0.238	− 0.037	0.879
*CRP/ACTB* expression ratio Digital drop PCR, N = 8, 10	− 0.024	0.051	− 0.142	0.639
hsCRP, N = 9,10	− 1.213	0.776	− 0.198	0.140

* N = number of samples from TT and CC individuals respectively.

** smoking defined as any reported use of tobacco.

## Discussion

Since a prior study found an association between the single nucleotide polymorphism, rs1205 and pre-eclampsia in this population^3^, our aim was to further ascertain the ability of leukocytes to express the CRP protein, and determine if such expression might be influenced by this SNP. Utilizing two different technologies and two disparate reference genes, the present study found very low relative expression of *CRP* mRNA harvested from peripheral leukocytes, on the order of 10^–4^ less *CRP* vs either of the reference genes. In addition, there was no evidence of differential expression dependent on the TT vs CC genotype.

Serum CRP has long been recognized as an important factor in the innate immune response^1^ and a measure of a more general inflammatory state which predicts future clinical disease^22–25^. Multiple *CRP* genetic variants are associated with these pathologic conditions, implying an in-born propensity. However, the evidence supporting a direct role of CRP as a primary causal agent, rather than acting in a contributory or modifying manner, remains controversial^11,26,27^. There is also countervailing evidence from Mendelian randomization studies, indicating no causal effects of increased serum CRP^28–30^.

A consideration in judging how CRP might be acting in a causal manner relates to the tissue(s) of origin. C-Reactive protein has been traditionally viewed as produced almost exclusively by the liver^6,13^. Although the predominant source of circulating CRP appears to be hepatic expression, there is documentation of production from other tissues, such as vascular^31^ and bronchial endothelia^32^, macrophages^33^, adipocytes^34^, renal cortical tubular epithelium^9^, neurons^10^ and vascular smooth muscle^35^, most often in contexts of local inflammation. The GTEx database^36^ found a median of 6,975 *CRP* transcripts per million, from 175 hepatic samples, only "outlier" values from pancreas and spleen, and no transcripts for whole blood. Of primary concern to the present study, the mRNA expression of *CRP* by leukocytes has been definitively reported^12,37^; but uncertainty remains as to whether this occurs to a significant extent in a non-stimulated state^9,15,38,39^.

A primary finding of the present study is very low expression of *CRP* mRNA (approximately 10^–4^ less than either reference gene), among unstimulated leukocytes. This finding is further supported by the fact that a pre-amplification PCR reaction was used to boost detection in the RT-qPCR method. Indeed, the calculated expression of *CRP* in all four of these methods is indistinguishable from the NRT control, suggesting that what was measured may simply be residual *CRP* genomic DNA, rather than mRNA. This is in spite of the fact that both the manufacturer gives assurance that the RNA extraction method eliminates most genomic DNA and an optional DNAse treatment was used. This finding is difficult to reconcile with results reported by Kaplan et al.^38^ showing ratios of *CRP* to *ACTB* of 0.7 from macrophages derived from THP-1 human monocytes induced by phorbol myristate acetate, although this cell line was induced to differentiate and exposed to other cell culture methods. Haider et al.^37^ also definitively reported *CRP* mRNA expression by unstimulated leukocytes from healthy subjects, albeit in absolute terms of ~ 5 pg / 2 × 10^7^ cells, and not relative to a reference gene. The present study is more in line with results of Yang et al.^15^ indicating barely detectable (density ratio ~ 0.05 compared with *GAPDH*) in unstimulated leukocytes from patients with stable angina pectoris. The overwhelming contribution of hepatic production compared to leukocyte expression also leads to the general lack of correlation between serum hsCRP and the leukocyte expression measures, with the exception of a negative correlation between the *GAPDH* digital drop ratio and hsCRP, which is difficult to explain.

The second finding is the lack of appreciable influence on leukocyte *CRP* expression due to the rs1205 genotype. This is perhaps understandable due to the very low mRNA expression levels discussed above, providing a weak signal against the background "noise". It seems that the great majority of circulating CRP derives from hepatic production and detecting any possible inherited genetic influences on leukocyte expression will likely require isolation of the leukocytes in cell culture. Multiple reports in the literature^16–18^ find an elevated hsCRP associated with the CC genotype, however our results show essentially equivalent mean hsCRP between the two genotypes. The further effects of this low signal-to-noise ratio are probably seen as well in the poor correlation between the 4 methods of determining mRNA expression reported here.

Strengths of this study include the use of two technical modalities of measurement (standard RT-qPCR and digital drop), as well as two different reference genes. The comparison between homozygous genotypes tends to enhance any potential differences in effect; and recruitment of females without a history of pre-eclampsia from a defined population would lessen environmental and background genetic variation.

The relatively small size of the cohort and the apparent, extremely low expression from this tissue reduced the study power and likely enhanced the effects of inherent assay variability, as discussed above. The need to pre-amplify the samples was also a likely contributor to assay variability.

## Methods

### Study recruitment and ethical approval

Approval was obtained from Institutional Review Boards of the Aberdeen Area Indian Health Service (16-A-01GP), the University of North Dakota (IRB-200207) and the Tribal Nations Research Group (protocol #39). Testing at Candeska Cikana Community College was done on anonymized samples, none of which derived from the local population. All work was done according to approved protocols. Written informed consent was obtained from each participant. All methods were carried out in accordance with relevant guidelines and regulations for the study.

Recruitment for the original case–control study has been ongoing from 2004 to the present; as previously described^40^. Pre-eclampsia case/control criteria were chosen to be compatible with the NIH Working Group on Research on Hypertension in Pregnancy (or "Working Group") definition^41^, while utilizing more stringent, repeat measures as recommended by both the Working Group and the American Society of Hypertension (ASH)^42^.

Controls of the parent study were ascertained by contact of individuals with dates of parturition closest prior and subsequent to the matching index case. The available medical records of all controls were abstracted in the same way as cases; and it was verified that these individuals did not meet criteria for pre-eclampsia (PE). The participants in the present study were re-contacted and re-consented a mean of 15.9 years after their delivery. To avoid influence of pathophysiologic sequelae of PE or other non-genetic risk factors associated with risk of PE, the present expression analyses were conducted only with control participants in apparent good health.

### Laboratory methods

Genotyping for the rs1205 SNP utilized TaqMan assays and primers (Life Technologies) on genomic DNA extracted from Oragene (DNA Genotek Inc) salivary samples.

Ten homozygous C and ten homozygous T controls were chosen; and early morning venipuncture samples obtained into EDTA tubes. Serum tubes were also drawn for simultaneous high sensitivity CRP (hsCRP) measurement. The RNA was extracted within less than two hours using the "QIAmp RNA Blood Mini" kit (Qiagen, Venlo, Netherlands). The optional DNAse digestion was conducted on the spin column using "RNAse Free DNAse" (Qiagen, Venlo, Netherlands). RNA concentration was determined using a NanoDrop 2000 (ThermoFischer Scientific, Waltham, MA) and samples standardized to 20 ng/μl.

Complementary DNA was then generated from the extracted RNA using iScript Reverse Transcription Supermix for RT-qPCR (Bio-Rad, Hercules, CA). Each reaction used 5 μl of 20 ng/μl RNA and standard quantities of supermix and water for a final volume of 20 μl. Next, the cDNA was "pre-amplified" using SsoAdvanced PreAmp Supermix (Bio-Rad, Hercules, CA) and PrimePCR PreAmp assay (Bio-Rad, Hercules, CA) for both *CRP* (Assay ID: qHsaCED0044459) and the *GAPDH* reference gene (Assay ID: qHsaCED0038674) sequences. Manufacturer recommendations for 10 μl of cDNA sample in a total volume of 50 μl were followed; and the resulting PCR products were diluted 1:5 in Tris–EDTA buffer as suggested.

Quantitative PCR was then carried out on the pre-amplified cDNA with SsoAdvanced Universal SYBR Green Supermix (Bio-Rad, Hercules, CA), using proprietary Bio-Rad primers PrimePCR *CRP* (qHsaCED0044459) and *GAPDH* (qHsaCED0038674). Expression qPCR runs again followed Bio-Rad recommendations with 1 μl primer, 7 μl water, 10 μl SYBR supermix and 2 μl of pre-amplified sample, for a total volume of 20 μl. Analysis was performed on 10 wells each of the *CRP* and *GAPDH* targets, in addition to a no template control (NTC) and a no reverse transcriptase (NRT) control well for each gene. In the same 48 well plate a standard dilution was run in duplicate with 6 wells progressively diluting *CRP* Bio-Rad template (Assay ID: qHsaCED0044459) and *GAPDH* template (Assay ID: qHsaCED0038674) in 1:5 steps from 2 × 10^6^/ul to a final 640 copies/ul. The unknown concentration of *CRP* and *GAPDH* cDNA was derived directly from the standard curve plotted on the Bio-Rad CFX Manager 3.1 software (Bio-Rad, Hercules, CA).

Expression analysis was also conducted on the same samples using Bio-Rad digital drop instruments and reagents. The robotic droplet generator, QX200 Auto DG Droplet Digital (Bio-Rad, Hercules, CA) performed the droplet generation for both the *CRP* and *GAPDH* targets, using Bio-Rad ddPCR GEX assay (Assay ID: dHsaEG5009107) for *CRP* and (Assay ID: dHsaEG5006642) for *GAPDH*. The volume of sample was 7.5 μl for *CRP* wells and 2.5 μl of 1:10 dilution for *GAPDH* wells, in addition to 10 μl QX200 ddPCR EvaGreen Supermix (Bio-Rad, Hercules, CA) , 1 μl of primer and water to a total of 20 μl well. Thermocycling parameters were per manufacturer's suggestion, with an annealing temperature of 58 °C. The Qx200 Droplet Reader (Bio-Rad, Hercules, CA) counted at least 17,000 validated droplets per sample to calculate copies per microliter, using the instrument generated threshold (Auto Analyze function of QuantaSoft Analysis Pro (1.0.596), unless a manual threshold call was required. Digital drop analyses with *GAPDH* controls were carried out at least twice for each sample and an additional 10 replicates among 7 samples. On each cartridge of 8 wells, one well was loaded with Bio-Rad template (as previously specified), the same volume as other samples; but at 1:1000 dilution for both *CRP* and *GAPDH*.

The same procedure was used for digital drop analysis of *CRP* using *ACTB* primer (Assay ID: dHsaEG5188254) as the reference gene. The same volumes and dilutions of sample were used as described above. Eight of the 20 samples were successfully replicated.

The serum hsCRP measures were done using a CRP Latex kit (Beckman-Coulter, Indianapolis, IN), an infrared particle immunoassay rate methodology at a local clinical laboratory. All samples were processed within 24 h of venipuncture.

The 2^ΔCT^ expression ratio was calculated as 2^(CT, reference gene—CT target gene)^ for the qPCR values, using the mean of the expression ratios of the 10 intra-run wells, excluding any values > 2 times the intra-run standard deviation (SD). The expression ratios derived from the standard curve were independently calculated from the individual wells that conformed to the same, within 2 SD of the intra-run mean criteria, as described above. The digital drop expression ratios were derived directly from the calculated concentrations of target and reference cDNA.

Samples with measured expression ratios beyond 1.5 X the interquartile range were excluded from analysis. In one instance, a replicate run was excluded due to a value at the extreme lower range of all samples and fivefold less than the earlier run on that same sample (which had shown a result well within the interquartile range).

### Statistical methods

Statistical analysis was conducted using SPSS version 13.0.1 software (IBM, Armonk, NY). Descriptive statistics report mean (+ /− SD) for continuous variables and proportions with 95% CI for discrete variables. Distributions were examined for normality using the Shapiro–Wilk test. Potential differences between the two groups with differing rs1205 genotypes were evaluated using the Student's t-test for continuous variables and the chi-square statistic for discrete variables. Linear regression was used to explore the multivariate association of genotype and other variables with the expression ratio of the target gene (*CRP*) with either of the two housekeeping genes (*GAPDH* and *ACTB*) individually. Statistical significance was set at *P* ≤ 0.05.

## Data availability

All data in the article can be requested from the corresponding author.
